# A comparative analysis of unhealthy food and beverage television advertising to children in Thailand, between 2014 and 2022

**DOI:** 10.1186/s12992-023-01007-7

**Published:** 2024-01-02

**Authors:** Nongnuch Jindarattanaporn, Bridget Kelly, Sirinya Phulkerd

**Affiliations:** 1 Institute for Population and Social Research, Mahidol University, Salaya Campus, 999 Phutthamonthon 4 Road, Phutthamonthon, 73170 Nakhon Pathom Thailand; 2 Early Start, School of Health and Society, Faculty of the Arts, Social Sciences and Humanities, University of Wollongong, Northfields Avenue, Wollongong, NSW 2522 Australia

**Keywords:** Advertising, Food, Beverage, Television, Child, Thailand

## Abstract

**Background:**

Food marketing is a key factor that influences children’s dietary behaviors. This study assessed the nature and extent of food and beverage advertising on television (TV) in 2014 and 2022 in Thailand.

**Methods:**

TV was recorded for one week in March 2014 and in May 2022 from 7-9am and 3-7 pm on weekends, and 3-7 pm on weekdays across two channels (64 h recorded each year). The nutrient profile model from Bureau of Nutrition, Ministry of Public Health Thailand was used to classify food and non-alcoholic beverages as: Group A (‘healthy’), Group B (‘less unhealthy’) or Group C (‘unhealthy’).

**Results:**

In 2014, 475 food advertisements were identified, with on average of 6.3 unhealthy food advertisements per hour. In 2022, 659 food advertisements were identified, with an average of 9.2 unhealthy food advertisement per hour. In both time periods, the most frequently advertised food products were non-alcoholic beverages. The rate of unhealthy food advertising per hour of broadcast was significantly higher than for other moderately unhealthy and healthy foods, and was also significantly higher in 2022 than in 2014.

**Conclusions:**

Food and beverage advertising on Thai television is predominantly promotes unhealthy foods and, in particular, sugar-sweetened beverages. Therefore, Thai Government should enact new legislation to protect children from food TV ads in order to control both the frequency and nature of unhealthy TV food marketing to protect the health of Thai children.

## Background

At least 2.8 million global deaths annually are attributable to overweight or obesity [1]. Worldwide, 39 million children under the age of 5 were overweight or obese in 2020 [2]. An estimated 340 million children aged five to nineteen years were overweight in 2016 [3]. Excess body weight in childhood places children at greater risk for becoming overweight and obese as adults, that later can put them at greater risk of non-communicable diseases [4, 5].

Childhood overweight and obesity remains a challenge in Thailand despite the Government’s commitment to childhood overweight and obesity prevention and management in national policies and plans. According to the Thailand Multiple Indicator Cluster Survey (MICS) and the national health examination survey, the prevalence of childhood overweight rose from 8.2% in 2015 to 9.2% in 2019 among Thai children aged under 5 years [6, 7]. The prevalence of childhood obesity rose from 5.3% in 1995 to 11.4% in 2014 among children aged 1–5 years and from 5.8% to 13.9% in the same period among children aged 6–14 years [8]. This health problem predominantly effects those with relatively high socioeconomic status [7, 9].

Food advertising is a key element of the food environment that can impact childhood obesity, by influencing children’s food preferences and choices, and dietary intakes [10–12].Exposure to food advertising increases unhealthy food and dietary energy consumption among children [13, 14]. In one randomised controlled trial conducted with Australian children, exposure to unhealthy food marketing on television and in a branded game led to an additional 194 kJ consumed across a subsequent snack and lunch meal, compared to when non-food products were promoted in these media [15, 16]. Over time, this energy surplus would be sufficient for the development of overweight in children [17].

Changes in the global food system have been associated with poor dietary patterns and obesity [18, 19]. Transnational food companies in the food retail, manufacturing and service sectors are dominant drivers of food system and dietary change in the Asia region [20]. Trade liberalisation and foreign direct investment has led to an increased presence of transnational food corporations in local markets, and greater availability, affordability, and consumption of processed Westernized [21]. In 2022, the food and beverage industries in Thailand spent US$491.5 million on advertising their products [22]. Three of the top 10 food advertisers were transnational companies, including Unilever Thai trading, Nestle Thailand and Coca-Cola Thailand [22].

Television (TV) advertising (ads) remains a major source of food marketing exposure for children [23], despite increasing investment and diversification in marketing media and techniques by food companies [24]. Most (94.2%) Thai households owned a television in 2020 [25] and the 2008 national mass media survey found that 99.5% of children watched television between 4 and 8 pm, and 99.9% were exposed to television on weekend mornings, between 4 to 10 am [26]. Previous studies from Thailand in 2010 and 2014, which monitored food advertising on television, found that the majority of food advertising was for unhealthy products [27, 28] and that the rate of unhealthy food advertising was stable across years [27, 28]. In a survey with Thai children in 2020, almost 90% of 10–14 year olds reported being exposed to TV advertisements in the past week for snacks, fried chicken, pizza and sugar-sweetened beverages, and more than 70% of 6–8 year olds were exposed to advertisements for food high in fat, sugar and salt, and sugar-sweetened beverages [29]. As a result, food industries advertise their products by targeting children’s television programmes [30].

There is evidence that restricting children’s exposure to unhealthy food and beverage advertising on TV is one of the most cost-effective interventions for Governments to improve obesity-related health outcomes for populations [31, 32]. In Thailand, earlier cost–benefit analyses have predicted that implementation of a policy to restrict children’s exposure to unhealthy food advertising on TV would reduce body mass index (BMI) in 6–12 year olds by an average 0.32 kg/m^2^ and would cost the Government 1.13 Million Baht for implementation. This was predicted to lead to a reduction in the prevalence of overweight and obesity children by 121,000 cases [29].

Currently, Thailand has some limited regulations to reduce the impact of food marketing, requiring snacks, desserts, semi-processed foods, frozen foods, bakery products, dairy products, and non-alcoholic beverages to display a warning message on the label and a warning message with clear sound and text for at least five seconds during advertisements on mass media (TV, radio and online) [33, 34]. In addition, presenters in TV advertisements, particularly for jelly products, must not be a medical or public health expert [35]. These regulations do not control the frequency of children’s exposure to unhealthy food marketing. Moreover, the National Broadcast and Telecommunications Commission that regulates broadcasting times for children’s, youth and family programs does not control food and beverage advertising at these broadcast times. The major barriers to policy implementation were a lack of a monitoring and evaluation system, a lack of organization knowledge regarding skills required for implementation, poor governance system, lack of funding and resources, lack of effective multi-sectoral platforms, influence of the food industry, lack of clear policy content, organizational culture and structure, and changes in policy priorities [36]. The Government of Thailand has recently directed efforts into new policies to restrict children’s exposure to unhealthy food and beverage marketing, in response to global recommendations for marketing controls from the World Health Organization [37]. The Department of Health at Ministry of Public Health Thailand has worked on drafting a Food Marketing Control Act, to restrict children’s exposure to unhealthy food marketing across media and settings [38].

Local data to describe the nature and extent of children’s exposure to food marketing are necessary for stimulating policy debate, informing government policies and actions, and for providing a basis for evaluating the effect of any ensuing marketing restrictions. However, the most available evidence on food and beverage TV advertising in Thailand was from 2014 [28]. The current study sought to update the available evidence on Thai children’s potential exposures to unhealthy food advertising on TV and identify if and how this advertising had changed over the past decade, to directly inform the policy debate for the Food Marketing Control Act.

## Methods

This was a repeat cross-sectional study that used comparable methods to capture television food advertising broadcast on the two most-watched TV channels in Thailand 2014 and 2022.

### Data collection

Each channel (Channel 3 is privately owned and the owner is BEC Multimedia Co., Ltd. and Channel 7 is privately owned and the owner is Bangkok broadcasting &TV Co., Ltd.) was recorded for seven days (five weekdays and two weekend days) from 7.00 to 9.00 am and 3.00 to 7.00 pm on weekend days (six hours/day/channel) and 3.00 to 7.00 pm on weekdays (four hours/day/channel). A total of 64 h television of broadcasting were recorded from March 24 to April 6, 2014 and from May 5 to 11, 2022. The broadcast times recorded were based on the programming times for children’s, youth and family programmes, as per the Notification of the National Broadcast and Telecommunications Commission [39]. All data were recorded from live television broadcasts onto DVDs.

### Data coding

All advertisements were coded in Microsoft Excel. The following information was collected for each advertisement: the channel name and number, date of recording, day of the week, program name and category, starting time of program, time slot of advertisement, advertisement type, company name, and food product category.

All food and beverage products were coded into two categories: 1 – products produced/sold by a transnational company, and 2—products produced/sold by a local company. This study defines ‘transnational company’ as an enterprise that is involved with the international production of goods, products or services, foreign investments, or income and asset management in Thailand. A local company is an enterprise for which the business and business owners reside in Thailand. Department of Business Development (DBD), Ministry of Commerce and company websites were searched to identify the company’s registration and any changes in ownership from 2014 and 2022 [40]. For each TV ad, the company’s product and name were coded.

Recordings were screened for food and beverage advertisements. Promoted food and beverage products were categorised using a nutrient profiling model developed by the Department of Health (DOH) [41]. This DOH nutrient profiling model was originally developed to assess the nutritional quality of food and beverage products consumed by Thai children aged 3–15 years. The model classifies foods into nine categories (snack food, desserts, semi-processed foods, processed foods, bakery products, cereals and grain products, and miscellaneous, dairy products, and nonalcoholic beverages) with threshold criteria for nutrients and ingredients (Table 1). Foods with an overall score of 8–10, 5–7 and 0–4 (out of a total score for each individual food category) were classified into Groups A (meeting standard, ‘healthy’), B (failing to pass in some criteria, ‘less healthy’) and C (failing to pass all threshold criteria, ‘unhealthy’).

**Table 1:** Nutritional thresholds for snack food, desserts, semi-processed foods, processed foods, bakery products, cereals and grain products, miscellaneous, dairy products, and nonalcoholic beverages [35]

	Nutritional thresholds for snack food, desserts, semi-processed foods, processed foods, bakery products, cereals and grain products, and miscellaneous					Nutritional content score of dairy products						Nutritional content score of nonalcoholic beverages				
	Energy (Kcal) per 100 g/ml	Fat (g) per 100 g/ml	Sugar (g) per 100 g/ml	Sodium (ml) per 100 g/ml	Food group (group)	Energy (Kcal)	Fat (g)	Sugar (g)	Protein (g)	Calcium (%)	Vitamin B2 (%)	Energy (Kcal)	Sugar (g)	Sodium (mg)	Ingredient (group)	Synthetic additives (number)
Total score for group A = 10	2	2	2	2	2	2	2	2	2	2	2	2	2	2	2	2
Group A (green) indicates healthy pass or meeting standard (‘healthy’)	≤ 100	≤ 3	≤ 6	≤ 100	> 3	≤ 120	≤ 7	≤ 10	≥ 7	≥ 30	≥ 25	≤ 40	≤ 10	≤ 40	1	0
Total score for group B = 5	1	1	1	1	1	1	1	1	1	1	1	1	1	1	1	1
Group B (yellow) indicates not meeting standard as failing to pass in some criteria (‘moderately unhealthy’)	101–200	4–6	7–12	101–200	2–3	121–144	7.1–8.4	10.1–12	5.6–6.9	24–29	20–25	41–80	11–20	41–80	2	1
Total score for group C = 0	0	0	0	0	0	0	0	0	0	0	0	0	0	0	0	0
Group C (red) indicates considerably under standard (‘unhealthy’)	> 200	> 6	> 12	> 200	1	> 144	> 8.4	> 12	< 5.6	< 24	< 20	> 80	> 20	> 80	3	> 2
'Food group' criteria refers to points given based on the number of recommended ingredients contained in the product, from the five core food groups in the Thai food-based dietary guidelines (rice, fruits, vegetables, meat and milk). For example, if a product contains fruits and meat, then it will be scored 1						-						Ingredient: (1) fruit and vegetables juices or herb containing 100% (2) fruit, vegetables or herb as mixture (3) no fruit and vegetables or herb Synthetic additives: synthetic color, preservatives, and sugar substitute				

Data coding was completed by three independent researchers in a two-step process. First, the first and second researchers each coded all data independently, and second, any discrepancies between these two researchers were reviewed by the third researcher and resolved by consensus among the three researchers.

### Data analysis

All analyses were conducted using SPSS for Windows version 22.0. Company’s advertising rates (the number of advertisements per hour, per channel, or per channel-hour) and frequencies were calculated by food category, weekdays and weekend days, and program categories. Poisson regression tests were used to test trends in food advertising rates across the two time periods. As the distributions of the outcome variables were highly skewed and had high zero counts (reflecting no advertising in some timeslots), further analyses using count models (Poisson, negative binomial, zero-inflated Poisson and zero-inflated negative binomial) were also examined. P-value less than 0.05 were considered to be significant.

## Results

### Food and beverage advertising trends between years

#### Overall

In 2014, 1,128 TV advertisements were identified across the two channels. The overall rate of food advertising was 7.4 advertisements per channel-hour. The overall rate of unhealthy food advertising was 6.3 advertisements per channel-hour, including 3.3 ads per channel-hour for unhealthy foods from transnational companies. Channel 7 had the highest rate of food advertising, at 7.8 advertisements per channel-hour (Table 2). In 2022, in the same number of broadcast hours, 2,529 advertisements were identified. The overall rate of food advertising was 10.3 advertisements per channel-hour. The rate of unhealthy food advertising was 9.2 advertisements per channel-hour, including 4.8 advertisements per channel-hour for transnational companies. Channel 3 had the highest rate of food advertising, at 13.7 advertisements per hour. Unhealthy food advertising dominated food advertisements on both channels. The ratio between healthy and unhealthy food advertising ranged from one healthy advertisement for every 20.3 unhealthy food advertisements in 2014, to one for every 13.4 unhealthy food advertisements in 2022.

**Table 2:** Mean rate of food advertisements by types of companies and food classification in 2014 and 2022

Channel	Total ads	Mean rate of food advertisements (ads per channel-hour) (n)											Ratio of healthy to unhealthy foods
		non-food	all food ads	All companies			Transnational companies			Local companies			
				Healthy foods	Less healthy foods	Unhealthy foods	Healthy foods	Less healthy foods	Unhealthy foods	Healthy foods	Less healthy foods	Unhealthy foods	
Year 2014	1,128	10.2 (653)	7.4 (475)	0.3 (20)	0.8 (50)	6.3 (405)	0.1 (9)	0.4 (26)	3.3 (212)	0.2 (11)	0.4 (24)	3.0 (193)	1:20.3
3	618	12.3 (393)	7.0 (225)	0.4 (12)	0.8 (26)	5.8 (187)	0.3 (8)	0.4 (12)	3.0 (95)	0.1 (4)	0.4 (14)	2.9 (92)	1:15.6
7	510	8.1 (260)	7.8 (250)	0.3 (8)	0.8 (24)	6.8 (281)	0.0 (1)	0.4 (14)	3.7 (117)	0.2 (7)	0.3 (10)	3.2 (101)	1:27.3
Year 2022	2,529	29.2 (1,870)	10.3 (659)	0.7 (44)	0.4 (27)	9.2 (588)	0.3 (20)	0.2 (13)	4.8 (368)	0.4 (24)	0.2 (14)	4.0 (220)	1: 13.4
3	1,261	25.8 (824)	13.7 (437)	0.7 (22)	0.5 (15)	12.5 (400)	0.4 (14)	0.3 (9)	7.9 (252)	0.3 (8)	0.2 (6)	4.7 (148)	1:18.2
7	1,268	32.7 (1,046)	6.9 (222)	0.7 (22)	0.4 (12)	5.9 (188)	0.2 (6)	0.1 (4)	3.6 (116)	0.5 (16)	0.3 (8)	2.3 (72)	1:8:5

Based on the ZIP regression analyses, the number of food advertisements in 2022 was significantly higher than in 2014 (β = 0.48, *p* = 0.001). Numbers of healthy food advertisements (β = 0.74, *p* = 0.011) and unhealthy food advertisements (β = 0.42, *p* = 0.011) in 2022 were significantly higher than in 2014.

There was a negative association between year of data collection and moderately or less unhealthy food advertisements. Number of moderately or less unhealthy food advertisements in 2022 was significantly lower than in 2014 (β = -0.85, *p* = 0.004). There was no statistically significant difference between weekday or weekend food advertising, and rate of unhealthy food advertisements (Table 3).

**Table 3:** Zero-inflated negative binomial regression analysis with outcomes variables

Variables	Number of food advertisements				Healthy food advertisements				Less unhealthy food advertisements				Unhealthy food advertisements				Rate of unhealthy food advertisements			
	β_i_	Sig	95% CI		β_i_	Sig	95% CI		β_i_	Sig	95% CI		β_i_	Sig	95% CI		β_i_	Sig	95% CI	
			Lower Bound	Upper Bound			Lower Bound	Upper Bound			Lower Bound	Upper Bound			Lower Bound	Upper Bound			Lower Bound	Upper Bound
(Constant)	2.78	0.000	-	-	-0.79	0.014	-	-	0.01	0.188	-	-	2.62	0.000	-	-	2.23	0.000	-	-
2014	1	-	-	-	1	-	-	-	1	-	-	-	1	-	-	-	1	-	-	-
2022	0.48	0.001	0.19	0.77	0.74	0.011	0.17	1.31	-0.85	0.004	-1.43	-0.28	0.42	0.008	0.11	0.73	0.08	0.530	-0.18	0.34
weekdays	1	-	-		1	-	-		1	-	-		1	-	-		1	-	-	
weekend days	0.12	0.429	-0.17	0.40	-0.30	0.318	-0.88	0.29	0.41	0.162	-0.17	0.99	0.10	0.543	-0.21	0.40	0.11	0.394	-0.15	0.38

*CI* refers to confidence interval

*β*_i_ refers to the parameter β (the regression coefficient) signifies the amount by which change in x must be multiplied to give the corresponding average change in y, or the amount y changes for a unit increase in x. In this way it represents the degree to which the line slopes upwards or downwards

#### Advertising on weekdays and weekends

Table 4 shows the mean rate of TV food and beverage advertisements on weekdays and weekends across years, by food classification. The mean rate of overall food advertisements and unhealthy food advertisements was higher in 2022 than in 2014, for both weekdays and weekends and for both channels. In 2014, the rate of unhealthy food advertisements was more than six times and five times higher than the combined number of healthy and moderately unhealthy food advertisements on weekdays and weekends, respectively. In 2022, the rate of unhealthy food advertising was almost nine times and eight times higher than the combined number of healthy and less healthy food advertisements on weekdays and weekends, respectively.

**Table 4:** Mean rate of TV food and beverage advertisements on weekdays and weekends by food classification in 2014 (*n* = 475) and 2022 (*n* = 659)

TV	Weekdays (Monday-Friday) (advertisements per channel-hour)			Weekend days (Saturday-Sunday) (advertisements per channel-hour)		
	Healthy foods	Less healthy foods	Unhealthy foods	Healthy foods	Less healthy foods	Unhealthy foods
Year 2014	0.4 (15)	0.7 (29)	6.9 (276)	0.2 (5)	0.9 (21)	5.4 (129)
3	0.5 (6)	0.9 (18)	6.1 (122)	0.3 (3)	0.7 (8)	5.4 (65)
7	0.3 (9)	0.6 (11)	7.7 (154)	0.2 (2)	1.1 (13)	5.3 (64)
Year 2022	0.8 (31)	0.3 (10)	8.9 (355)	0.5 (13)	0.7 (17)	9.7 (233)
3	0.9 (18)	0.5 (10)	13.8 (276)	0.3 (4)	0.4 (5)	10.3 (124)
7	0.7 (13)	0.0 (0)	4.0 (79)	0.8 (9)	1.0 (12)	9.1 (109)

#### Advertised food and beverage product categories

Overall, non-alcoholic beverage products had the highest rate of advertising in 2014 and 2022, and were promoted an average of 4.5 times per hour in 2022 (Table 5). Most advertisements for non-alcoholic beverages promoted unhealthy products. Non-alcoholic beverage products of both transnational companies and local companies contributed the highest rate of advertising in 2014 and 2022. In 2014, the top three products with the greatest number of advertisements were non-alcoholic beverages (33.3% of all advertisements, n158), processed foods (19.6%, n93) and dairy products (16.4%, n78). In 2022, the top three advertised food products were again non-alcoholic beverages (44.2%, n291), dairy products (19.6%, n129) and miscellaneous products (14.9%, n98, including supplements and condiments).

**Table 5:** Mean rate of food advertisements by food category and types of companies

Food and beverage category	Mean rate and percentage of food advertisements (ads per channel hour)																					
	Total of food advertisements (n)		All companies Mean rate of advertisements (ads per channel hour)		All companies Mean rate of advertisements (ads per channel hour)						Transnational companies Mean rate of advertisements (ads per channel hour)						Local companies Mean rate of advertisements (ads per channel hour)					
	2014	2022	2014	2022	2014			2022			2014			2022			2014			2022		
					Healthy foods	Less healthy foods	Unhealthy foods	Healthy foods	Less healthy foods	Unhealthy foods	Healthy foods	Less healthy foods	Unhealthy foods	Healthy foods	Less healthy foods	Unhealthy foods	Healthy foods	Less healthy foods	Unhealthy foods	Healthy foods	Less healthy foods	Unhealthy foods
Overall	475	659	7.4	10.3	0.3	0.8	6.3	0.7	0.4	9.2	0.1	0.4	3.3	0.3	0.2	5.8	0.2	0.4	3.0	0.4	0.2	3.4
1. Non-alcoholic beverages	158	291	2.5	4.5	0.1	0.0	2.3	0.5	0.0	4.0	0.0	0.0	1.1	0.2	0.0	2.0	0.1	0.0	1.3	0.3	0.0	2.0
2. Processed foods	93	28	1.5	2.0	0.0	0.2	1.3	0.0	0.1	2.0	0.0	0.1	0.7	0.0	0.0	0.4	0.0	0.1	0.6	0.0	0.0	0.0
3. Dairy products	78	129	1.5	2.0	0.0	0.1	1.1	0.2	0.2	1.1	0.0	0.0	0.6	0.0	0.0	1.6	0.0	0.1	0.5	0.0	0.1	0.4
4. Snacks	37	40	0.6	0.6	0.0	0.0	0.6	0.0	0.0	0.6	0.0	0.0	0.1	0.0	0.0	0.5	0.0	0.0	0.5	0.0	0.0	0.1
5. Miscellaneous (supplements and condiments)	32	33	0.5	0.5	0.2	0.1	0.2	0.0	0.0	0.5	0.1	0.1	0.1	0.2	0.2	0.4	0.1	0.0	0.1	0.1	0.0	0.8
6. Semi-processed foods	31	28	0.5	0.4	0.0	0.0	0.5	0.0	0.0	0.4	0.0	0.0	0.4	0.0	0.0	0.5	0.0	0.0	0.1	0.0	0.0	0.1
7. Desserts	26	19	0.4	0.3	0.0	0.3	0.2	0.0	0.1	0.2	0.0	0.2	0.2	0.0	0.0	0.1	0.0	0.0	0.0	0.0	0.0	0.1
8. Bakery products	20	14	0.3	0.2	0.0	0.1	0.2	0.0	0.0	0.2	0.0	0.0	0.2	0.0	0.0	0.2	0.0	0.1	0.0	0.0	0.1	0.0
9. Cereals and grain products	0	7	0.0	0.1	0.0	0.0	0.0	0.0	0.0	0.1	0.0	0.0	0.0	0.0	0.0	0.1	0.0	0.0	0.0	0.0	0.0	0.0

#### Advertising patterns across the day

In 2014, the highest rate of unhealthy food advertisements was observed from 6-7 pm on weekdays, with almost 21.7 unhealthy food advertisements shown during this 1 h period (Table 6). On weekend days, the highest rate of unhealthy food advertisements was found from 8-9am (14.5 advertisements/hour). In 2022, the highest rate of unhealthy food advertisements was reported from 3-4 pm (14.0 advertisements/hour) on weekdays, followed by the time slot 6-7 pm (9.1 advertisements/hour). On weekends the highest rate of unhealthy food advertising occurred between 7-8am (14.5 advertisements/hour) and 8-9am (11.8 advertisements/hour).

**Table 6:** Mean rate of TV food advertisements by time slot

TV	Total of food advertising	Mean rate of food advertisements					
		Weekdays (Monday-Friday)			Weekend days (Saturday-Sunday)		
		Healthy foods	Less healthy foods	Unhealthy foods	Healthy foods	Less healthy foods	Unhealthy foods
Year 2014	475	0.4 (15)	0.7 (29)	6.9 (276)	0.2 (5)	0.9 (21)	5.4 (129)
7:00–7:59	36	-	-	-	0.3 (1)	1.8 (7)	7.0 (28)
8:00–8:59	71	-	-	-	0.5 (2)	2.8 (11)	14.5 (58)
15:00–15:59	38	0.1 (1)	0.2 (2)	1.7 (17)	0.0 (0)	0.5 (2)	4.0 (16)
16:00–16:59	12	0.0 (0)	0.0 (0)	1.2 (12)	0.0 (0)	0.0 (0)	0.0 (0)
17:00–17:59	66	0.2 (2)	0.4 (4)	3.0 (30)	0.5 (2)	0.3 (1)	6.8 (27)
18:00–18:59	252	1.2 (12)	2.3 (23)	21.7 (217)	0.0 (0)	0.0 (0)	0.0 (0)
Year 2022	659	0.8 (31)	0.3 (10)	8.9 (355)	0.5 (13)	0.7 (17)	9.7 (233)
7:00–7:59	64	-	-	-	0.8 (3)	0.8 (3)	14.5 (58)
8:00–8:59	54	-	-	-	1.0 (4)	0.8 (3)	11.8 (47)
15:00–15:59	191	0.9 (9)	0.7 (7)	14.0 (140)	0.5 (2)	0.5 (2)	7.8 (31)
16:00–16:59	110	0.6 (6)	0.0 (0)	6.4 (64)	0.3 (1)	1.0 (4)	8.8 (35)
17:00–17:59	104	1.1 (11)	0.0 (0)	6.0 (60)	0.3 (1)	0.3 (1)	7.8 (31)
18:00–18:59	136	0.5 (5)	0.3 (3)	9.1 (91)	0.5 (2)	1.0 (4)	7.8 (31)

## Discussion

This study is the first to determine Thailand trends in food and beverage advertising in major broadcasting TV channels, and to compare the rate of food and beverage advertising by company, weekends and weekdays, time slots, and nutrition quality across two major TV channels over time. This study also investigated the association between years of data collection and rate of food and beverage advertisements. The study highlights three major findings: TV remains a major food advertising medium, unhealthy food products dominate TV food advertising, and unhealthy food advertisements are concentrated during the broadcast times that attract the greatest numbers of child viewers. Hence, unhealthy food advertising exposure among children has worsened in Thailand over time.

Transnational companies identified in this study, particular Coca Cola (Thailand) Company Limited, Nestle Thailand Company Limited and Pepsi Co (Thai) Trading were signatories to the Thailand Children’s Food and Beverage Advertising Initiative (Thai Pledge) [42] and International Food & Beverage Alliance (IFBA) Global Responsible Marketing Policy [43, 44]. Despite these commitments, transnational companies had higher rate of food advertising than local companies both in 2014 and 2022, and transnational companies had higher rate of unhealthy food advertising than local companies on both two channels in 2022. These industry pledges are, clearly, ineffective in limiting unhealthy food advertising on television, a finding that has also been documented elsewhere [45].

TV remains a major advertising medium for food and beverage products in Thailand. This might be due to two channels of TV are privately owned and have the highest ratings or the most viewers in Thailand [46]. Therefore, there are many food advertisements. Our results showed that the rate of food advertisements rose from 7.4 ads per channel-hour in 2014 to 10.3 ads per channel-hour in 2022. Current food marketing regulations in Thailand do not control the frequency of food advertisements or the types of foods that can be promoted [33–35]. Advertising expenditure of the food and beverage industries in Thailand rose from US$24.5 million per year in 2014 to US$491.5 million per year in 2022 [22, 47]. The results are not consistent with findings from other countries, which have found a reduction in TV food advertising over time. For example, in one Australian study the frequency of television food advertising was significantly lower in 2009 and 2011 compared to 2006 [48]. This likely reflected a shift in marketing efforts to digital platforms and also the increased scrutiny of industry marketing practices at the time [43].

The contributions of unhealthy food and beverage products to food advertising were substantial and collectively dominated food advertisements, especially non-alcoholic beverage products. Similar trends have been observed in Asia–Pacific countries [49–51]. Further, in Thailand the rate of unhealthy food advertising was found to have increased significantly over time. In contrast, legislation in Korean to control children’s exposure to unhealthy food advertising on TV between 5:00 and 7:00 pm [52], has led to a decline in unhealthy food advertising. During the four-month period following the implementation of the Korean Law, the number if unhealthy food advertisements decreased from 1,296 in 2009 to 243 in 2010 [53]. An evidence from policy evaluations is limited for the outcomes of changes to population diets. However, such policies have been found to lead to reduced purchases for unhealthy foods and also reduced exposure to unhealthy food marketing [54]. Moreover, evidence indicates that there is a clear association between exposure to unhealthy food advertisements on TV and increased food consumption and calorie intake [55, 56]. This association may lead to increase the prevalence of overweight and obesity among children [55]. The present study suggests that the frequency of food and beverage advertisements and the nutritional quality of promoted foods should be restricted in Thai regulations. The rates of advertising for unhealthy foods are a conservative estimate, given that the DOH nutrient profiling model used to classify foods does not consider the use of artificial sweeteners or other sugar substitutes in the evaluation criteria [41]. This limitation is similar to the Mexico’s nutrient profiling model [57]. In contrast, foods with non-nutritive sweeteners are not permitted to be marketed to children according to the WHO South-East Asia Region (SEARO) nutrient profiling model [58]. As such, foods containing no sugar, but artificial sweeteners or other sugar substitutes were evaluated with a high score based on the sugar threshold. Modifications to the DOH model should be considered prior to its application in food policies, to ensure it aligns with other nutrition recommendations and authoritative nutrient profiling models for classifying foods not permitted to be marketed to children.

Advertising for moderately or less healthy foods decreased over time, from 0.8 ads per hour in 2014 to 0.4 ads per hour in 2022. This change was a result of small reductions in advertising for ‘less healthy’ dessert items, bakery products, processed foods and miscellaneous foods. While this change was statistically significant, it represents very small changes in the actual rates of advertising for these foods; such as a reduction in the rate of advertising for desserts from 0.3 ads/hour to 0.1 ads/hr. These small reductions in moderately unhealthy food advertising do not detract from the large increase in advertising rates for unhealthy foods over time.

TV advertising of unhealthy food and beverage products appeared most frequently during broadcast periods with the greatest numbers of child viewers. The rate of unhealthy food advertising was highest on weekend days, when Thai children spend an average of three hours per day watching TV [59]. The timeslots with the highest rates of unhealthy food advertising corresponded to periods of the highest child viewership. Rates of unhealthy food advertisements were highest on weekends from 7-9am and on weekdays between 4-7 pm. Similarly, research from Malaysia and the United States found that a majority of unhealthy food products advertisements was concentrated on Saturday morning programs, and there was lower advertising of healthy food items compared to weekdays [51, 60]. However, according to the national Mass Media survey 2008, Thai children watched in greater numbers outside of these children’s, youth and family programmes. 99.5% of children aged 6–14 years watched television between 4 and 8 pm, and 99.9% were exposed to television on weekend mornings, sometime between 4 to 10 am [26]. Therefore, the food advertising control should be based on times when Thai children are watching and not just when it is these designated program times.

The decline of food advertisement rate was observed in certain food categories. The rate of processed food advertisements, particular fast-food advertising, on Thailand TV in 2022 was lower than in 2014. In addition, in 2022 the times with the highest number of advertisements have a lower rate per hour compared with 2014. This may be explained by a shift from traditional advertising to digital advertising. According to AC Nielsen Thailand data, fast food companies invested more on advertising online (+ 7.50%) than on TV (-1.55%) in Y2022 [22]. Online marketing expenditures for fast food increased from 24,766 million baht in 2021 to 26,623 million baht in 2022, while advertising expenditure on TV declined from 63,649 million baht in 2021 to 62,664 million baht in 2022 [22]. Worldwide, 71% of youth (ages 15–24 years) are online and children and adolescents under 18 accounted for an estimated one in three internet users globally in 2017 [61]. As advertising expenditure shifts online, there is a need for monitoring to understand the nature and extent of children’s food marketing exposures on this media.

This study has limitations that should be acknowledged. Firstly, the narrow sample periods used are a limitation, although the periods were comparable between years. The patterns observed for these sample time periods may not reflect advertising across the entire year. Second limitation in the design of this study is that channels of TV in 2014 and 2022 were not chosen to cover children’s peak viewing times since this information was not easily available, nor affordable to access. In addition, TV was not recorded over 24 h. However, the national survey confirmed that the majority of children and youth were exposed to television on weekend mornings (4–10 am) and weekday late afternoons to evening (4–8 pm) and this corresponded with the broadcast periods captured in this study [26].

## Conclusions

This study assessed the rate and nutritional profiles of food and non-alcoholic beverage advertising on Thai television. The results support policy stakeholders, such as governments and public health advocates, to determine the best approach for controlling children’s exposures to unhealthy food and beverage marketing in Thailand. The findings suggest that unhealthy food and beverages dominate TV advertising and rates of advertisements are highest during the broadcast periods with the greatest numbers of child viewers. Current regulations to control unhealthy food advertising on TV are inadequate and must be adapted urgently and strengthened to protect children from the impacts of this marketing. The new regulation should control the frequency of unhealthy food advertising during children’s, youth and family programmes on weekdays and weekend.

## Data Availability

This study analyzes quantitative data. The coding sheet and quantitative analysis table are available from the corresponding author on reasonable request.
